# Nutritional Significance of Fruit and Fruit Products in the Average Polish Diet

**DOI:** 10.3390/nu13062079

**Published:** 2021-06-17

**Authors:** Krystyna Rejman, Hanna Górska-Warsewicz, Joanna Kaczorowska, Wacław Laskowski

**Affiliations:** Department of Food Market and Consumer Research, Institute of Human Nutrition Sciences, Warsaw University of Life Sciences, 07-787 Warsaw, Poland; krystyna_rejman@sggw.pl (K.R.); joanna_kaczorowska@sggw.pl (J.K.); waclaw_laskowski@sggw.pl (W.L.)

**Keywords:** fruit and products, energy sources, fiber sources, bananas, apples, sources of vitamins, sources of minerals, sources of carbohydrates

## Abstract

The aim of the present study was to analyze the supply of energy, 19 nutrients, free sugars, and dietary fiber in the average Polish diet from fruit and fruit products. Our analysis is based on 2016 data from the national representative household budget survey conducted on a sample of 36,886 households, yielding a population of 99,230 individuals. Fruit and fruit products provided 3.12% of energy to the average diet in Poland with the highest share of bananas and apples. The highest significance of this food group was found for vitamin C (23.65%), including citrus fruits providing 8.03% of vitamin C, berries (5.97%), other fruits (3.45%), and apples (3.13%). The share of fruit and their products in the supply of free sugars is equally high and amounts to 23.52%. This means that apples provide 6.34% of free sugars, while other fruits also have a high supply of free sugars, including berries 3.68%, stone fruits 3.06%, bananas 2.56%, and citrus fruits 2.54%. The proportion of supply exceeding the percentage of energy (which was 3.12%) was obtained for carbohydrate (5.79%), and fiber (13.66%). The food group studied was particularly important (more than 5% share) in providing four minerals: potassium (8.59%), iron (5.07%), magnesium (5.51%), copper (8.81%), and three vitamins: vitamin C (23.65%), vitamin B6 (5.74%), and vitamin E (5.53%). The influence of sociodemographic and economic characteristics of households on the structure of energy and nutrient supply from fruit and fruit products was assessed using cluster analysis. There were four clusters characterized by different energy, nutrient, and fiber supply. The factors with the highest statistical significance on the supply of energy, nutrients, and fiber from fruit and fruit products were month of study, income, degree of urbanization, education, size of town, and land use. The obtained results concerning energy and nutrient supply from fruits and fruit products are important for the Polish society from the public health point of view, as indicated in the discussion of results and conclusions.

## 1. Introduction

It is recognized that fruit and fruit products consumption is associated with protective effects against various chronic diseases [1], particularly cardiovascular diseases [2,3], hypertension [4], type 2 diabetes [5], different types of cancer [2,6,7,8], asthma [9], obesity [10], cognitive impairment [11], and depression [12,13]. Fruits and vegetables are the cornerstone for a healthy and sustainable diet i.e., with low environmental impacts which contribute to food and nutrition security, and to healthy life for present and future generations [14]. According to various expert bodies, the greatest benefits to human and planetary health can be achieved by consuming a minimum of 400 g of fruit and vegetables per person each day, which in dietary practice means five servings including a higher proportion of vegetables [15,16,17,18]. However, due to a number of factors, such as availability, affordability or lack of knowledge and awareness, the typical daily fruit intake among many people around the world is far below the recommended level [19,20]. 

In a botanical sense, the term ‘fruit’ refers to the edible part of a plant that is a mature ovary, consisting of seeds, covering, and any closely connected tissue without any consideration of whether these are edible. Essentially, culinary fruits are the subset of botanical fruits that remain after the exclusion of cereal grains, nuts, seeds (including pulses), and fruits used as vegetables (avocado, cucumber, tomato, pumpkin, eggplant, etc.) [21]. Nuts (walnuts, hazelnuts, almonds, pistachios, peanuts, cashews, etc.) are perceived as a special type of dry fruits that are distinguished by their woody shell and have a different nutritional value [22]. Fruits can be divided into several groups such as stone fruits (cherries, plums, peaches, apricots, nectarines), pome fruits (apples, pears), berries (blackberry, blueberry, cranberry, raspberry, strawberry), citrus (oranges, mandarin, tangerine, grapefruit, lemon), melons (watermelon, cantaloupe, casaba, honeydew melon), “Mediterranean” fruits (grapes, fig, kiwi, dactyls), tropical fruits (banana, pineapple, mango, pomegranate, papaya), and others [21,23].

Depending on the type, variety, and origin as well as the way they are grown, harvested, and stored, fruits vary greatly in color, texture, flavor, health properties, and nutritional value. Fresh fruit consists mainly of water, which can make up to 90% of its total weight. This makes them perishable and results in high susceptibility to spoilage after harvest. It is estimated that up to 50% of fruit produced in developing countries are lost in the supply chain between harvest and consumption [24]. To extend the shelf life, fruits are processed into various fruit products, such as frozen foods, canned food, juices, nectars, jams, preserves, etc. Nevertheless, processed fruits have a reduced health and nutritional value, because the preservation process negatively affects the content of nutrients and bioactive substances [25].

Approximately 50–80% of the total dry matter of fruits are carbohydrates, usually in the form of simple sugars (glucose, fructose, and sucrose) that contribute to a sweet taste. The exception is the carbohydrates contained in bananas, classified as resistant starch, which functions similarly to dietary fiber [26].

The dietary fiber content of fruit is typically between 1% and 3%, except for nuts and dried fruit, where it is much higher, e.g., in almonds 12%, walnuts 7%, prunes 12%, raisins 4% [26]. Predominant fiber components are pectin, hemicellulose and cellulose which have a prebiotic effect (which helps boost immune systems), regulate satiety, prevent gut and lung cancers, reduce the risk of cardiovascular disease and type 2 diabetes [20].

In general, fruits have low lipids and proteins content. However, nuts are a rich source of high-quality proteins and unsaturated fatty acids, which can regulate a wide range of functions including blood pressure, blood clotting, blood lipid levels, the immune and the inflammatory responses [22].

Many species of fruits are rich in vitamin C (especially berries and citrus fruits), carotenoids (cherries, apricots, melons, mangoes), and vitamins E and B (nuts), which all have important antioxidant potential [27]. Furthermore, they are also a good source of polyphenolic compounds (catechins, anthocyanins, isoflavones, resveratrol, quercetin, genistein, etc.) that exhibit antiproliferative, antidiabetic, anticancer, antimicrobial, anti-inflammatory, antiviral, and antioxidant properties. Significant amounts of these bioactive substances can be found in frequently consumed fruits, such as berries, citrus, grapes, apples, and cherries [28,29].

Overall fruit and their products are rather poor in minerals, but some of them are relatively rich in calcium, iron, and copper (berries), phosphorus (apricots, peaches, bananas, nuts), magnesium and potassium (bananas) or zinc, for example bananas, pecans, and walnuts. These micronutrients found in fruit may be protective for bone health, possibly because of their alkaline-forming properties. The epidemiological studies have confirmed positive links between fruits and vegetables consumption and bone health in adolescents and older adults [30,31].

Knowing these arguments, the aim of the present study was to analyze the supply of energy and nutrients in an average Polish diet from the total group of fruit and fruit products and from individual types. Our analysis is based on 2016 data from the representative household budget survey. This is another study on understanding the importance of specific food groups as sources of energy and nutrients in the average Polish diet. To date, research has been published on the nutritional importance of the following food groups: meat, meat products and seafood [32], cereals and cereal products [33], milk and dairy products [34], nonalcoholic beverages [35], butter, margarine, vegetable oils and olive oils [36], vegetables, potatoes and their products [37]. Our study also presented food sources of protein and amino acids [38] and potassium [39] in the average Polish diet.

## 2. Materials and Methods

### 2.1. Study Description

The aim of our study was to analyze sources of energy and nutrients to the average diets in Poland from fruit and fruit products, divided into nine product subgroups. We presented the contribution of energy, macronutrients (protein, fat, carbohydrates), fiber, minerals, and vitamins. We presented the results in Section ‘Results’ in the following order:

Supply of energy, macronutrients, and dietary fiber.
Supply of minerals i.e., calcium, phosphorus, sodium, potassium, iron, magnesium, zinc, and copper.
Supply of vitamins, i.e., thiamine, riboflavin, niacin, folate, vitamins: B_6_, C, A, and E.
Supply of energy and nutrients according to household characteristics.

### 2.2. Data Collection and Analysis

Our study is based on data from the national representative Household Budget Survey (HBS). The survey was conducted by national statistical office Statistics Poland, which ensures representativeness by drawing households in a two-stage procedure (first research areas, then households) and allows generalizing the results to the entire population of Poland. In 2016, the survey was conducted on a sample of 36,886 households, yielding a population of 99,230 individuals [40]. This method of obtaining information on consumption levels is being improved [41,42,43,44]. The details of the technical way of conducting the survey, including the sampling and randomization method, the method of supervision, how to record information in the “Household Budget Diary” and “Household Statistics Sheet”, how to record the amount of food items consumed have been presented in our previous studies [32,33,34,38].

The unit data present the monthly consumption of 91 food items in each household. The data have been converted into energy, 19 nutrients and dietary fiber using the latest edition of the Polish food composition and nutritional value tables [45]. The next step was to calculate average values for energy and nutrient supply, first in weight units and then in percentage terms. The final step was to determine the effect of economic and sociodemographic factors on energy and nutrient supply. We publish calculations for specific food categories because of their nutritional importance to public health. The research process involved seven steps and is described in one of our previous publications [34]. For free sugars only, we adopted the WHO (World Health Organization) definition and the calculation method derived from the difference between total and individual carbohydrate supply [46].

We used R program v 3.0.2 (Copyright (C) 2018, The R Foundation for Statistical Computing, Vienna, Austria), a statistical computing system and environment including matrix and vector operations [47,48], to recalculate intake data from 36,886 households for 99,230 individuals across 91 food subgroups and energy, nutrients and dietary fiber. For a more detailed analysis, we determined the influence of 14 demographic, social, and economic factors on the level and structure of energy and nutrient supply from fruit and fruit preparations. Among the factors considered are education, income (quintile group), socioeconomic affiliation of the household, degree of urbanization, size of village or city, region, stage of family life, land use, self-assessed material situation, self-assessed nutritional status, age, gender, month of participation in the study, and number of persons in the household. The influence of individual factors was determined by the Cramer correlation index. We conducted cluster analysis [49,50] and identified four clusters for the study households using the Neural Networks module in Statistica 13.3 (Copyright 1984-2917, TIBCO Software Inc., Palo Alto, CA, USA) and Kohonen Neural Network [51]. For the four clusters, we presented the characteristics of the factors for which the value of the Cramer correlation index was significant (*p* < 0.05).

### 2.3. Food Grouping

Data from the HBS survey allowed us to identify nine generic subgroups of fruit, nuts, and fruit products. When making this division, we did not consider the botanical classification, but the customary perception of fruits by consumers and their place in Polish eating habits [44,52]. These are:

(1) citrus fruits—lemons, oranges, mandarins, grapefruits, pineapples;
(2) bananas;
(3) apples;
(4) berries—strawberries, raspberries, blueberries, cranberries, white-, red- and blackcurrants, blackberries, gooseberries, wild strawberries;
(5) stone fruits—plums, cherries, sour cherries, apricots, peaches, nectarines;
(6) other fruits—for example, mangoes, kiwis, grapes, figs, dactyls, papayas;
(7) frozen fruits—all single types and mixed;
(8) dried fruits and nuts—all kinds of dried fruit, nuts, including salted and roasted nuts, almonds, edible seeds (e.g., poppy seeds, pumpkin seeds, sunflower seeds);
(9) fruit products—pasteurized fruit, canned fruit, pickled fruit, fruit salads.

## 3. Results

### 3.1. Supply of Energy, Macronutrients, and Dietary Fiber

In the energy value of the average daily food consumption in Poland, equal to 2261 kcal per person, fruits and fruit products contributed 70.5 kcal (Table 1). Their share was therefore 3.1%. The two species, apples, and bananas had almost identical and largest shares of 0.6% each, and the third source was the dried fruit and nuts subgroup, contributing just over 0.5%. Frozen fruits and fruit products were of minimal importance in providing energy.

Carbohydrates were the main macronutrient provided in the diet by the food group. Almost 16 g of carbohydrates, or 5.8% of the total dietary supply of this nutrient, came from fruit and fruit products. The supply of the other two macronutrients at about 1 g per day (and 1% contribution) was virtually negligible. As with energy, apples and bananas stood out in carbohydrate provision at nearly 1.3%, but citrus fruits came in third (0.7%). Two more subgroups—berries and nectarines—contributed more than 0.5%. Some importance of dried fruits and nuts was revealed in the provision of fat and protein, as they had 0.6% and 0.4% of their daily supply from the analyzed food group. 

Dietary fiber is the food component that determines the role of fruit in a healthy diet. Their presence in the Polish average diet means an intake of 2.4 g of fiber per day per person and this is 13.7% of the total fiber intake. Apples and berries stand out in providing fiber with more than 3%, and citrus fruits and dried fruits and nuts stand out with nearly 2%.

### 3.2. Supply of Minerals

The data in Table 2 show that fruit and fruit products do not provide sodium, a major risk factor for high blood pressure, one of the most common noncommunicable diseases (NCDs). The nutritional importance of fruits and processed fruits shows the significant contribution of this food group in providing copper and potassium, 8.8 and 8.6%, respectively, and over 5% in providing magnesium (5.5%) and iron (5.1%). Citrus fruits were the first source of copper and calcium, and third for potassium, magnesium, iron, and phosphorus. Bananas were the first source of potassium (2.1%) and magnesium (1.7%). Apples, on the other hand, made a large contribution (second source) in providing three minerals: potassium, iron, and zinc. Berries stood out for their contribution in providing iron (1.0% and first place), copper, phosphorus, zinc, and calcium. Additionally, of note was the subgroup of dried fruits and nuts. Due to low water content in their composition, they were distinguished by a significant share in the supply of six minerals: phosphorus, iron, zinc (first place), magnesium and calcium (second place), and copper (third place).

### 3.3. Supply of Vitamins

The place of fruit and fruit products in the diet is determined by their high vitamin C content. In an average Polish diet, they provided 21.6 mg/person of this vitamin, which meant 23.7% share of its total dietary intake (Table 3). Each subgroup contributed to the vitamin C supply, among them citrus fruits (8.0%), berries (6.0%), and a group of other fruits (3.5%) stood out. Fruit and fruit products also contributed significantly to vitamin B_6_ (5.7%), vitamin E (5.5%), and folate (4.9%). Vitamin B_6_ came primarily from bananas, citrus fruits, apples, and berries, vitamin E came from dried fruits and nuts, apples, and berries, and folate came equally from citrus fruits, bananas, berries, and a group of other fruits (e.g., mangoes, kiwis, grapes, figs, dactyls, papayas). The importance of the dried fruit and nut subgroup is less than that of the mineral supply because environmental parameters like temperature and time are not allies of the vitamins.

### 3.4. Supply of Energy and Nutrients According to Household Characteristics

To determine the influence of economic and sociodemographic characteristics of the household on the level and structure of energy and nutrient supply from fruit and fruit products, we conducted a cluster analysis. First, we determined that month of study, income expressed in quintile groups, degree of urbanization, level of education, socioeconomic affiliation of household, size of the town, and land use were the factors with the greatest influence on the supply of energy and nutrients from fruit and fruit products (Table 4). We then divided the study population into four clusters based on *p* < 0.05. Table 5 shows the characteristics of the clusters including the criteria with the highest significance, at *p* < 0.05.

**Figure 1:**
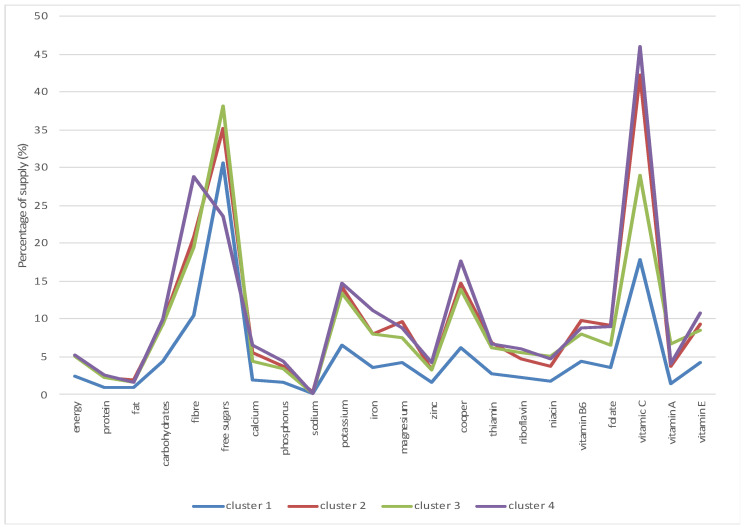
Cluster analysis: supply (in %) of energy and nutrients from fruit and fruit products.

The most numerous cluster (cluster no. 1) is characterized by the lowest energy supply from fruit and fruit products (2.4% with a mean value for the entire study population of 3.12%) (Table 6, Figure 1). The supply of energy from fruit and fruit products in the remaining clusters is above 5%. The supply of vitamin C varies from 17.78% in cluster no. 1 to 45.92% in cluster no. 4 with the average value of 23.65%. The supply of free sugars varies from 18.61% in cluster no. 1 to 38.05% in cluster no. 4 (mean value for the whole study population 23.52%). Additionally, in the case of fiber the lowest supply was recorded in cluster no. 1 (10.41%), the highest in cluster no. 4 (28.77%) with the average value of 13.66%. 

## 4. Discussion

In this section, we compare our results showing the nutritional importance of fruit, nuts and processed fruit products in the average diet in Poland with the corresponding results of dietary studies in other countries (United States [53,54], Australia [55], New Zealand [56], UK [57], The Netherlands [58], and Spain [59]). It is worth noting here that some limitations in these comparisons result from differences in methodological assumptions of research on average food consumption in individual countries. The discrepancies concern the different classification of food groups and separation of population groups. We then discuss the results obtained in the context of dietary guidelines and public health.

### 4.1. Energy, Free Sugars, and Dietary Fiber

Our study indicated that fruit and fruit products contributed 3.12% of energy to the average diet in Poland, with apples (0.63%), bananas (0.62%), and dried fruits and nuts (0.55%) contributing the most. Similarly, in the average US diet for adults ≥ 51 years, fruit and products provided 3.17% of energy [53]. The average data for the whole US population is lower indicating that fruit contributes 2.3% of energy [54]. In the average Australian diet, a similar result was obtained for men. Fruit products and dishes provided 3.0% of energy for men and 4.4% of energy for women, with pome fruit contributing the most (0.8% and 1.2%) [55]. Higher results for fruit energy supply were obtained in the case of the Dutch and New Zealand diets. According to the Dutch National Food Survey fruits, nuts and olives provided 5% of energy [58], whereas in the average New Zealand diet according to the New Zealand Adult Nutrition Food Survey fruits provided 5.4% and nut seeds 1.2% [56]. A slightly lower energy value for fruit was reported in the average English diet; according to results from 7/8 combined program of National Diet and Nutrition Survey, fruit provided 3.6% and nuts and seeds 1.5% of energy in the average diet of people aged 19–64 years. However, in the 65–74 and over 75 age group, fruits provided 4.6% and 3.8%, respectively, while nuts and seeds provided 1.3% and 1.1% [57]. 

In this set of results two groups of countries clearly stand out. In the first one there were Poland, US, and Australia, where the share of energy from the analyzed food group was 3.0–4.4%. Only Poland included nuts in this food group. In the second group of countries with a higher share of fruit, nuts and vegetables in dietary energy were the Netherlands, New Zealand, and England. New Zealand is the record holder here, with a percentage of 6.6%. However, the Dutch diet also included olives, a fruit unique for its high fat content, 15 g in 100 g of product, in this food group [60]. However, it is nuts among the subcategories discussed that bring fat into the diet. The fat content per 100 g of nuts ranges from less than 50 g for pistachios, cashews, and peanuts (44.4, 46.4, and 49.2, respectively) to more than 70 g for macadamia nuts and pecans (75.8 and 72.0) [61]. After vegetable oils, nuts are the second richest plant food in fat. Furthermore, the fatty acid composition of nuts is beneficial to health, as the content of saturated fatty acids is low, ranging from 7.4% of total fatty acids in hazelnuts to 22.7% in brazil nuts. The same is true for protein as nuts are also an excellent source of protein (approximately 25% of their energy value). In the average Polish diet, the subgroup of dried fruit and nuts provides the most protein in the analyzed food group. 

The provision of energy from fruit and fruit products is therefore linked to the content of carbohydrates, especially free sugars. In the average Polish diet, these products provided 5.79% of carbohydrates, including apples (1.29%), bananas (1.29%), and citrus fruits (0.72%). Higher carbohydrate intake from fruit and fruit products was reported in Australian, English, Dutch, and New Zealand diets. In the average Australian diet, fruit products and dishes provided 6.1% of carbohydrates for men and 8.6% for women, including pome fruit (1.8% and 2.5%, respectively) and tropical fruit 1.8% and 2.8% [55]. In the UK average diet of people aged 19–64 years, fruit provided 6.5% and nuts and seeds 0.4%. In contrast, in the diets of older people aged 65–74 and over 75 years, the proportion of fruits was 8.3% and 7.1%, and nuts and seeds was 0.2% for both age groups [57]. In the average Dutch diet, fruits, nuts and olives contributed 6% of the carbohydrate supply [58], while in the average New Zealand diet, fruits contributed 8.9%, and nuts and seeds contributed 0.3% [56]. Only in the case of the American diet, the share of fruit in the carbohydrates supply was shaped at the level of 4.8% [54]. Compared to the New Zealand diet, this is almost twice the proportion.

The importance of fruit and fruit products in nutrition is shown by their 13.66% share of dietary fiber in the average Polish diet, with apples (3.2%), berries (3.1%), citrus fruits (1.9%), and dried fruits and nuts (1.9%) contributing the most. In all comparable diets, fruits and processed fruits provided less fiber. For example, in the average American diet, fruit provided 10.2% [54], whereas in the diet of people over 51 years, fruit provided 11.7% [53]. Similar values were obtained for the average Dutch diet, according to the Dutch National Food Survey fruit, nuts and olives provided 11% of fiber [58]. For New Zealand, higher values were obtained, fruit provided 11.5% of dietary fiber, while nut and seeds provided 1.3% [56]. Lower values for fiber from fruit and fruit products were indicated in the average Australian diet. Fruit products and dishes provided 10.6% fiber for men and 13.8% fiber for women, including pome fruit (3.3% and 4.2%, respectively), citrus fruit (1.5% and 1.8%), stone fruit (1.1% and 1.6%), tropical fruit (2.5% and 3.4%), other fruit (0.9% and 1.3%) [55]. In the average English diet of 19–64 year old’s, fruit provided 8.4% of fiber and nuts and seeds provided 1.8%, in the diet of 65–74 year old’s these values were 11.6% and 1.6%, and in the diet of 75+ year old’s these values were 9.2% and 1.4% [57].

The almost 14% share of the analyzed food group in the provision of dietary fiber in the Polish diet was determined by the generic structure of consumption and the important place of apples in the dietary traditions and fruit production in Poland. Apples are eaten in the largest quantity of any fruit species consumed in households. They are a good source of fiber, containing on average 2 g of fiber in 100 g, including 1.5 g of soluble fiber, mainly pectin [45]. Citrus fruits also provide fiber in the form of pectin, while nuts and berries are mainly insoluble fiber. Nuts are a nutrient-dense food and a good source of dietary fiber. Its content ranges from 4 to 11 g per 100 g, and in standard serving satisfy 5–10% of the daily fiber requirements [61].

### 4.2. Minerals and Vitamins

Potassium, iron, magnesium, and copper are minerals provided in the average Polish diet by the analyzed food group in an amount exceeding 5% of the total intake. 

Fruit and fruit products provided potassium in the average Polish diet at level of 8.59%, with bananas contributing the most, at 2.13%, apples (1.43%), and citrus fruits (1.29%). Higher values were recorded in the average New Zealand diet, with fruits providing 9.8% of potassium and nut seeds providing 0.9% [56]. In the Australian diet potassium was contributed by fruit products and dishes at 7.0% (men) and 9.6% (women), including pome fruit (1.2% and 1.6%, respectively), and tropical fruit (2.5 and 3.5%) [55]. In England, on the other hand, fruit provided 7.2% of the potassium in the average diet of 19–64-year old’s, 8.6% in the diet of 65–74-year old’s, and 7.1% in the diet of those over 75. Nuts and seeds provided potassium at 1.2%, 1.0%, and 0.8%, respectively [57]. A lower potassium supply than in Poland was reported in the average Dutch and American diet. According to the Dutch National Food Survey, fruits, nuts, and olives contributed 7% of potassium [58], whereas in the US 5.8% in the average diet of adults [54] and 7.18% in the diet of adults ≥ 51 years [53]. A similar level of supply as for potassium from fruit and fruit products was recorded for copper. Fruit and fruit products provided 8.81% of the copper, with citrus fruits (1.6%), berries (1.57%), dried fruits and nuts (1.52%), and bananas (1.27%) contributing the most. Of the diets analyzed, only the Dutch diet reported copper supply. Fruits, nuts, and olives provided 9% of the copper [58].

Iron in the average Polish diet was provided by fruit and fruit products in the amount of 5.07%, including berries—1.01%, apples—0.82%, and citrus fruits—0.67%. Only in the average New Zealand diet fruit and nut seeds provided a total of 5.6% iron [56]. The other comparable diets had lower values: 4% of iron was provided by fruits, nuts and olives according to the Dutch National Food Survey [58], 1.72% in the total US diet, adults ≥51 years [53], and 2.9% and 4.0% of iron from fruit products and dishes in the average Australian diet of men and women [55]. Differences were also noted in a nationally representative sample of the Spanish population: fruit provided 4.6% in men’s and 5.7% in women’s diets [59]. In the average English diet of people aged 19–64 years, fruit contributed 2.9% of the iron, while nuts and seeds provided 1.5%. For those aged 65–74 and over 75, these values were 4.0% and 1.3% and 3.8% and 1.2%, respectively [57]. 

In the average Polish diet, fruit and fruit products provided 5.51% of the total magnesium supply. Higher values were obtained in the Dutch National Food Survey, where fruits, nuts and olives accounted for 7% of magnesium supply [58]. On the other hand, according to the UK National Diet and Nutrition Survey, fruits provided 5.3% (19–64-year-olds), 6.1% (65–74-year-olds), and 5.2% (75+-year-olds) of magnesium, respectively. Nuts and seeds, on the other hand, accounted for a supply of 3.1%, 2.3%, and 2.2%, respectively [57]. A lower magnesium supply was found in the average diet of Americans over 51 years of age (4.2%) [53] and Australians. According to the Australian National Food Survey, fruit products and dishes provided 3.7% of magnesium in the diet of men and 5.1% in the diet of women, including tropical fruit (1.4% and 2.1%) [55].

The nutritional significance of fruit and fruit products in the Polish diet is also related to the provision of vitamins. Here, vitamin C is the most important, with its share of almost 25%, as well as vitamins B_6_ and E with less than 6%. Citrus fruits (8.0%), berries (6.0%), other fruits (3.5%), and apples (3.1%) contributed the most to the vitamin C supply. All the diets included in the comparison had a lower vitamin C intake from fruit and fruit products. For example, in the average Spanish diet, fruit provided 20.05% of vitamin C [62], in total US diet, adults ≥ 51 years—18.22% [53], and in the New Zealand diet 22.4% [56]. According to the Dutch National Food Survey, fruits, nuts and olives provided 16% of vitamin C [59], and according to the Australian National Food Survey, fruit products and dishes accounted for a supply of 16.9% of vitamin C in the diets of men and 21.1% in the diets of women, including pome fruit (1.5% and 1.8%), citrus fruit (7.6% and 8.3%), tropical fruit (3.4% and 4.5%), and other fruit (2.3% and 3.6%) [55]. 

The supply of vitamin B_6_ from fruit and fruit products in Poland was 5.53% with the highest contribution from bananas (2.78%). In an average Dutch diet, fruit contributed 12.5% and nut seeds 0.6% [56], whereas in an average Spanish diet fruit provided 8.8%. [63]. A comparable vitamin B_6_ supply from the group of fruit and fruit products was obtained in the total US diet, adults ≥ 51 years—5.92% [53]. 

Vitamin E from fruit and fruit products was provided to the average Polish diet in the amount of 5.53%, the largest shares were provided by apples (1.19%), and dried fruits and nuts (1.45%). Two studies reported lower vitamin E intake, i.e., 4.81% in the average Spanish diet [62] and 2.63% in the total US diet, adults ≥ 51 years [53]. However, in the average Dutch diet, fruits, nuts and olives provided 6% vitamin E [59], whereas in the New Zealand diet, fruits provided 7.2% vitamin E, nuts and seeds 2.4% [56].

Comparing the results of our own study with the nutritional value of diets in other countries did not show a clear differentiation, as the contribution of a food group to the nutritional value of a diet depends on the consumption pattern, both in terms of quantity and variety.

### 4.3. Fruit and Fruit Products in the Polish Diet and Public Health Issues

The cluster analysis showed that more than 2/3 of the population was in Cluster no. 1 with the lowest consumption of fruit and fruit products. This group was dominated by households with low and average income (66%), the largest number of households (almost half) represented the two lowest levels of education, and the largest number in relation to the other clusters were white-collar workers, farmers, and recipients of social benefits. The consumption pattern in Polish households is strongly determined by income situation and sociodemographic characteristics [64,65,66]. The coefficient of income elasticity of fruit consumption is high, so an improvement in the material situation of households would contribute to an increase in fruit consumption [67,68]. Data from 2016 HBS [40] show that fruit and fruit products consumption in the fifth income group of households (the second determinant in the cluster analysis) is 2.3-fold higher than in the first quintile group. By assortment group, the differences range from 3.6 times for processed fruits, 2.8 times for berries, 2.5 times for citrus fruits and bananas (together) to 1.6 times for apples. The third factor was the degree of urbanization, and, in this case, consumption was highest in the largest cities, 40% higher than in the villages, where it was paradoxically lowest.

In the households of Cluster no. 1, fruit is eaten mainly in autumn and winter when it is relatively cheap, but even so, the average daily intake of all analyzed nutrients, energy and fiber was lowest here. The highest indices generally applied to households from Cluster no. 4 (high income and education level), and the differences between these clusters in the intake of nutrients from fruit and its products were more than double or even 3-fold (calcium and iron). In these households (also from Cluster no. 3) fruit consumption dominates in the spring and summer months, which was related to the appearance of various types of berries on the market in the following months.

A survey among young adult Poles (18–35 years old, equal proportions of both sexes) showed that almost half eat fruit at most once a day and only 7.5% eat it 4–5 times a day. It also found that men ate significantly less fruit and fewer vegetables than women [69]. The results of the national survey on fruit and vegetable consumption conducted in 2020–2021 [70] showed that almost all respondents (98%) know that vegetables and fruit are worth eating. Half of those surveyed know their health benefits (they provide vitamins, minerals, and antioxidants), 37% appreciate their taste and know that they are the basis of a healthy diet, and 30% believe they have a beneficial effect on health, well-being, and appearance. At the same time ¾ do not know how much fruit and vegetables one should eat. Half of the respondents eat very little fruit and vegetables because the stereotype that preparing fruit and vegetables is difficult and time-consuming is repeated. Most respondents have a specific set of vegetables and fruit that must be at home. It is worth noting, however, that vegetables and fruit are often bought on impulse, when they appeal or are spotted while shopping. Fruit and vegetables are rarely treated as a main meal to which other ingredients are chosen. Studies have shown that meals eaten throughout the day contain practically no dishes in which fruit and vegetables are the leading ingredient. The opposite is true—they are usually only a decorative addition and may even be absent.

These facts reflect the two main characteristics of the consumption of fruit and fruit products in Poland—low levels and high seasonality. For years, Poland has had a kind of fruit paradox, with very high production and very low consumption. Our country is the largest producer of apples in the EU and the third largest in the world (after China and the USA). We also lead Europe in the production of sour cherries, some berry fruits (currants, raspberries, gooseberries), and we are second in the production of strawberries and blueberries. With a share exceeding 50%, Poland is also the leading producer of frozen fruit and concentrated juices from soft fruit [71]. Despite this high fruit production, consumption has for years remained at one of the lowest levels in the EU. The average monthly household consumption was 3.66 kg/person, or 122 g/person per day, including 36 g of apples, 19 g of bananas, and 22 g of citrus fruit (in 2016). This is therefore on average 1.5 portions, calculated according to the generally accepted standards suggested by the WHO. Consumption data at the level of food balances are not optimistic either—in 2016 it was 54 kg per year [72], or an average of 148 g per day, which amounts to 1.85 portions. This includes the additional amount of fruit eaten outside the home (in food service sector), but also the loss and waste of fruit in the supply chain. In 2019, balance data show a 7% increase (58 kg/person or 159 g/person/day), achieving two portions of fruit available for consumption. With this level of consumption, Poland is at the bottom of the EU countries. Fruit and nuts consumption is higher in all countries whose data we confront with the results of our analysis. The FAOSTAT database [73] in 2016 for Poland reports a fruit supply of 56.61 kg/person and a nut supply of 0.96 kg/person. The highest supply of fruit was reported in the Netherlands, 110.24 kg/person, and nuts in Spain, 7.14 kg/person.

The largest seasonal variations (own calculations, 2016 data) are found in the consumption of berries: the relative amplitude of variation (showing the spread of consumption limits) was 35.4, the average relative deviation (showing how many percent each month of the year deviates from the monthly average) was 79%. Slightly lower variations are found in the consumption of citrus fruits (ratios of 8.9 and 61%, respectively) and apples (3.2 and 21%), and the lowest for bananas (1.4 and 8%). The occurrence of seasonality in the generally low intake of fruit means that the supply of vitamins, especially vitamin C and bioactive compounds from fruit may be inadequate during the autumn and winter months. The role of these foods in the provision of vitamin C cannot be overestimated. In the Polish diet it is the second source of this vitamin (23.7%), after vegetables with preserves (37.7%) and before potatoes (14.1%) [37]. Vitamin C must be supplied with food, because due to the lack of L-gulonolactone oxidase in humans, the synthesis of L-ascorbic acid is not possible. In addition, it is one of the most labile vitamins, sensitive to increased temperature, oxygen, certain enzymes, and metal ions [74]. Representative population studies do not provide data on the intake of non-nutritive bioactive compounds contained in fruit, which show beneficial effects on many human body functions. The state of current scientific knowledge gives strong evidence that eating fruit and vegetables, as well as wholegrains and fiber, play a crucial role in protecting against certain cancers, as well as weight gain, overweight and obesity [75].

The health monitoring of British adults examined the associations between dietary fiber intake and sources from food and four measures of body composition (body mass index, body composition, waist circumference, C-reactive protein). Fruit fiber was the only source of fiber that was consistently inversely associated with all measures of body composition, while whole grain sources of fiber were inversely associated with three, and vegetables (including pulses) with two [76]. In the average Polish diet, fruit is the third source of fiber (13.7% of total intake), after cereal products (48.5%) [33] and vegetables (22.7%) and before potatoes (9.1%) [37]. The fiber content of food is rather low, so it must be collected from a wide variety of dietary fiber sources, both relatively high-fiber and low-fiber foods [77]. Recommendations for adults in different countries indicate a fiber intake of 18–38 g/day, and WHO/FAO [78] in its recommendations for the world population set the intake at 25 g/day. Fiber in the Polish diet is a deficient component (17.6 g/person/day according to our calculations), which is common in highly developed countries.

The demand to increase fruit consumption for health reasons may bring the risk of increasing the intake of free sugars from fruit and fruit products. Current recommendations focus on limiting the intake of free sugars, which include sugars naturally present in honey, syrups, fruit juices, and fruit juice concentrates, together with monosaccharides and disaccharides added to foods and beverages by the manufacturer, cook or consumer [79]. Excessive consumption of fructose in humans and animals have been associated with adverse metabolic effects. However, only some fruits, pears, and apples, are rich in fructose. Fructose, as commonly consumed in mixed-carbohydrate sources, does not exert specific metabolic effects that could account for weight gain [80]. The consumption of fruit is sometimes wrongly associated with a risk of overweight or obesity, since eating fruit is essential for providing beneficial micronutrients, fiber, and many bioactive compounds with high antioxidant activity. The health benefits argue for an increase in fruit consumption, especially those low in free sugars, and a reduction in fruit juices, which can make up one portion of the five recommended. 

Among the dietary mistakes most frequently made by Poles, too little intake of vegetables, fruit, and nuts is indicated. That is why, among others, the dietary guidelines for the Polish population have undergone significant changes in 2020, in terms of graphics (changing the pyramid to a plate of healthy nutrition) and content [81]. It is recommended that vegetables and fruit should constitute half of the weight of food consumed per day (half of the plate). For the first time, it has been quantitatively indicated that 400 g of fruit and vegetables is the daily minimum, with more vegetables than fruit, and the more the better. The health benefits of eating different colored fruit and vegetables were also highlighted. For health and environmental reasons (the first verbal reference to environmental aspects) it was recommended to eat pulses and nuts instead of meat. Therefore, in our opinion, nuts and seeds should be a separate group in the HBS methodology so that changes in their consumption can be tracked.

In the context of the high proportion of free sugars from fruit and fruit products in the current diet, it should be noted that the recommended increase in fruit intake should be one of many changes in dietary structure. In the context of public health, both dimensions of diet—quantitative and qualitative—are important.

### 4.4. Strengths and Limitations of Our Study

The advantage of our research is the sample size of 2016 HBS (the largest research sample in Poland), the highest number of listed products and product groups (91), constant approach to the methodology implemented for many years and improved, as well as representative sample selection. Household surveys are conducted using a random sample within a representative survey method that allows for generalizability of the results. The HBS records monthly consumption; each household participates in the survey and records the quantity (and cost) of all food products obtained for consumption in the household for a period of one month.

However, there are some limitations in the survey methodology. The self-recording of consumption information in the diary can lead to under and/or overestimation of the data, even though HBS has well-developed procedures to control all records. Controls have increased in recent years due to the move to an electronic-only system for monitoring consumption and making records. Another limitation may relate to the quality of the food consumption table. We used the current edition of the “Food Composition and Nutritional Value Tables” (2017) as a nutritional database to convert 91 products and product groups into energy and nutrients. This version of the tables is significantly revised, incorporating new products and technological modifications. This may affect the difficulty in comparing current results with data from earlier years. In addition, our calculations are based on household food consumption and do not consider eating out.

The limitations of survey methods listed above are common and typical. However, household budget surveys are the only such comprehensive and representative method of collecting data on food consumption and other living conditions of the Polish population. Owing to this and the analyses of subsequent food groups undertaken, our results may serve to develop or verify dietary recommendations and educational programs from the point of view of public health.

## 5. Conclusions

Poland is a country with an under-consumption of fruit, both in terms of dietary recommendations and health benefits, and in relation to its harvest and supply. Despite this, our research has shown the importance of fruit and fruit products, including nuts, in the average Polish diet. They are the second source of vitamin C and the third source of dietary fiber, and provide over 5% of some minerals (potassium, iron, magnesium, copper), as well as vitamin B_6_ and vitamin E. Eating fruit of any kind, regardless of taste preferences, has health significance in the prevention of chronic diet-related diseases. The greater the variety, the higher the health benefits. Therefore, current dietary guidelines in many countries, including Poland, focus on plant-based diets to eat complex carbohydrate-containing foods, including grains, vegetables, fruits, pulses, nuts, and seeds to provide the body with fiber, micronutrients, and bioactive compounds. Our research has revealed important barriers to increasing fruit consumption. These are mainly the seasonality of supply, which affects the seasonality of fresh fruit consumption and household income. This knowledge is worth using in programmes to promote an increase in the level and variety of fruit consumption. An appropriate message, including culinary fruit workshops, should be targeted at all population groups.

## Figures and Tables

**Table 1:** Supply of energy, macronutrients and dietary fiber from fruit and fruit products.

Specification	Energy	Protein	Fat	Carbohydrates	Fiber	Free Sugars
Average Daily Supply	2261.00 kcal	77.90 g	96.91 g	270.37 g	17.64 g	30.70 g
Daily Supply from Fruit and Fruit Products Share (%) in Average Daily Diet	70.54 kcal	1.03 g	1.10 g	15.65 g	2.41 g	7.22 g
	3.12%	1.32%	1.13%	5.79%	13.66%	23.52%
List of Fruit and Fruit Products by Supply (in %) ^1/:^						
Citrus fruits	0.34	0.17	0.04	0.72	1.90	2.54
Bananas	0.62	0.18	0.05	1.27	1.43	2.56
Apples	0.63	0.15	0.13	1.29	3.19	6.34
Berries	0.31	0.17	0.06	0.66	3.05	3.68
Stone fruits	0.28	0.13	0.04	0.54	0.92	3.06
Other fruits	0.26	0.10	0.14	0.46	1.06	2.39
Frozen fruits	0.01	0.00	0.00	0.01	0.07	0.07
Dried fruits and nuts	0.55	0.39	0.64	0.63	1.89	2.67
Fruit products	0.11	0.02	0.03	0.21	0.15	0.22

^1/^ 100% is taken to be the total energy or nutrient supply in the diet, e.g., citrus fruits provide 0.34% of the total energy in an average Polish diet (0.34% of 2261 kcal).

**Table 2:** Supply of minerals from fruit and fruit products.

Specification	Calcium	Phosphorus	Sodium	Potassium	Iron	Magnesium	Zinc	Copper
Average Daily Diet	644.10 mg	1160.19 mg	3863.84 mg	2617.85 mg	10.28 mg	267.33 mg	9.78 mg	1.11 mg
Daily Supply from Fruit and Fruit Products Share (%) in Average Daily Diet	18.48 mg	25.29 mg	5.41 mg	224.87 mg	0.52 mg	14.73 mg	0.21 mg	0.10 mg
	2.87%	2.18%	0.14%	8.59%	5.07%	5.51%	2.12%	8.81%
List of Fruit and Fruit Products by Supply ^1/:^								
Citrus fruits	0.81	0.30	0.01	1.29	0.67	0.77	0.20	1.60
Bananas	0.13	0.24	0.00	2.13	0.55	1.69	0.26	1.27
Apples	0.19	0.22	0.02	1.43	0.82	0.32	0.46	1.01
Berries	0.59	0.34	0.01	1.01	1.01	0.57	0.33	1.57
Stone fruits	0.27	0.20	0.01	0.84	0.47	0.33	0.15	0.95
Other fruits	0.20	0.16	0.01	0.77	0.35	0.43	0.16	0.71
Frozen fruits	0.01	0.01	0.00	0.02	0.02	0.01	0.01	0.03
Dried fruits and nuts	0.61	0.69	0.01	0.99	1.03	1.32	0.52	1.52
Fruit products	0.05	0.02	0.07	0.11	0.14	0.06	0.03	0.14

^1/^ 100% is taken to be the total supply of each mineral in the diet, e.g., citrus provides 0.81% of the total calcium in the average Polish diet (0.81% of 644.10 mg).

**Table 3:** Supply of vitamins from fruit and fruit products.

Specification	Thiamin	Riboflavin	Niacin	Vitamin B_6_	Folate	Vitamin C	Vitamin A	Vitamin E
Average Daily Diet	1.32 mg	1.59 mg	16.21 mg	1.84 mg	275.02 µg	91.40 mg	1.194.55 µg	13.45 mg
Daily Supply from Fruit and Fruit Products Share (%) in Average Daily Diet	0.05 mg	0.05 mg	0.40 mg	0.11 mg	13.39 µg	21.62 mg	29.15 µg	0.74 mg
	3.75%	3.15%	2.46%	5.74%	4.87%	23.65%	2.44%	5.53%
List of Fruit and Fruit Products by Supply ^1/:^								
Citrus fruits	0.76	0.29	0.19	0.61	1.14	8.03	0.41	0.77
Bananas	0.45	0.88	0.47	2.78	1.14	1.50	0.11	0.34
Apples	0.74	0.47	0.31	0.47	0.62	3.13	0.11	1.19
Berries	0.44	0.40	0.25	0.47	0.73	5.97	0.08	0.82
Stone fruits	0.40	0.36	0.40	0.28	0.22	1.06	0.75	0.48
Other fruits	0.35	0.26	0.30	0.56	0.67	3.45	0.49	0.37
Frozen fruits	0.01	0.01	0.01	0.01	0.01	0.17	0.00	0.02
Dried fruits and nuts	0.56	0.44	0.49	0.51	0.32	0.22	0.43	1.45
Fruit products	0.05	0.04	0.04	0.04	0.03	0.11	0.05	0.11

^1/^ 100% is taken to be the total supply of each vitamin in the diet, e.g., citrus fruits provide 0.76% of the total thiamine in the average Polish diet (0.76% of 1.32 mg).

**Table 4:** Cluster analysis: impact of sociodemographic and economic factors on the supply of energy and nutrients from fruit and fruit products in the average Polish diet.

Factors	Cramer Correlations
Study month	**0.418**
Income (quintile group)	**0.113**
Degree of urbanization	**0.084**
Education level	**0.080**
Socioeconomic affiliation	**0.078**
Size of the town	**0.077**
Land use	**0.076**
Number of people in a household	0.066
Assessment of the financial situation	0.060
Family life phase	0.055
Gender	0.047
Assessment of nutrition	0.044
Age	0.040
Region	0.037

The most important factors are written in bold.

**Table 5:** Cluster analysis: characterization of clusters in terms of designated socioeconomic characteristics.

Specification	Sample Population	Cluster 1	Cluster 2	Cluster 3	Cluster 4
	100%	67.1%	14.1%	10.1%	8.7%
Number of households	36,886	24,758	5208	3724	3196
Factors	% of sample population	% of households in the cluster (100%)			
Study month					
Month: 01	8.4%	8.3%	19.7%	0.1%	0.2%
Month: 02	8.4%	9.2%	15.5%	0.2%	0.3%
Month: 03	8.4%	9.4%	14.3%	0.1%	0.3%
Month: 04	8.3%	10.7%	6.3%	0.4%	2.1%
Month: 05	8.3%	10.4%	2.5%	1.0%	10.1%
Month: 06	8.4%	5.8%	0.7%	8.7%	40.6%
Month: 07	8.4%	6.0%	0.6%	31.5%	12.2%
Month: 08	8.3%	5.9%	0.7%	31.6%	11.9%
Month: 09	8.3%	7.3%	0.6%	21.3%	13.2%
Month: 10	8.3%	10.3%	2.5%	4.5%	7.0%
Month: 11	8.4%	9.7%	11.7%	0.5%	1.9%
Month: 12	8.3%	7.1%	25.0%	0.1%	0.2%
Income (quintile group)					
1st group	20.0%	23.9%	11.4%	12.5%	12.6%
2nd group	20.0%	21.5%	15.6%	17.8%	17.9%
3rd group	20.0%	20.2%	18.7%	21.1%	19.4%
4th group	20.0%	18.5%	24.4%	21.8%	22.5%
5th group	20.0%	15.9%	29.9%	26.7%	27.7%
Degree of urbanization					
Densely populated area	35.4%	31.8%	44.2%	42.3%	40.8%
Medium populated area	22.9%	22.9%	23.6%	22.5%	21.9%
Sparsely populated area	41.7%	45.3%	32.2%	35.2%	37.3%
Education level					
Lower secondary, primary	13.7%	15.5%	9.5%	10.3%	11.4%
Basic vocational	31.3%	33.7%	24.8%	29.2%	25.5%
Secondary and post-secondary	32.6%	31.7%	34.5%	33.2%	36.1%
Higher	22.4%	19.3%	31.2%	27.3%	27.0%
Socioeconomic affiliation					
White-collar workers	24.5%	27.1%	19.1%	20.5%	17.6%
Employees in manual labor positions	24.0%	21.8%	30.9%	26.9%	26.3%
Farmers	4.6%	5.3%	2.1%	3.8%	4.0%
Self-employed	6.8%	6.3%	8.0%	7.6%	7.4%
Pensioners	29.9%	28.4%	31.1%	33.3%	34.8%
Retired	6.3%	6.5%	5.5%	5.4%	6.6%
Recipients of social benefits	2.6%	3.1%	1.4%	1.5%	2.0%
Living from other unearned sources	1.5%	1.5%	1.8%	1.3%	1.4%
Size of the town					
500 thousand residents and more	12.9%	11.1%	17.5%	15.8%	16.1%
200–499 thousand residents	8.5%	7.4%	11.0%	10.5%	10.6%
100–199 thousand residents	7.9%	7.5%	8.7%	9.2%	8.2%
20–99 thousand residents	17.2%	16.6%	19.2%	18.6%	17.3%
Less than 20 thousand residents	11.2%	11.2%	11.9%	10.8%	10.9%
Village	42.3%	46.3%	31.6%	35.1%	37.0%
Land use					
Yes	52.6%	54.8%	44.0%	49.8%	53.2%
No	47.4%	45.2%	56.0%	50.2%	46.8%

Values above the sample population averages are written in red.

**Table 6:** Cluster analysis: supply (in %) of energy and nutrients from fruit and fruit products.

Specification	Sample Population	Cluster 1	Cluster 2	Cluster 3	Cluster 4
Energy	3.12	2.40	5.06	5.04	5.27
Protein	1.32	0.95	2.22	2.19	2.55
Fat	1.13	0.92	1.97	1.66	1.57
Carbohydrates	5.79	4.48	9.55	9.25	9.99
Fiber	13.66	10.41	20.70	19.51	28.77
Simple sugars	23.52	18.61	30.54	35.22	38.05
Calcium	2.87	2.00	5.59	4.33	6.46
Phosphorus	2.18	1.58	3.73	3.46	4.44
Sodium	0.14	0.11	0.22	0.16	0.15
Potassium	8.59	6.50	14.28	13.35	14.70
Iron	5.07	3.64	7.94	7.94	11.17
Magnesium	5.51	4.17	9.69	7.49	8.78
Zinc	2.12	1.61	3.32	3.20	4.27
Cooper	8.81	6.27	14.74	13.92	17.72
Thiamin	3.75	2.73	6.79	6.12	6.66
Riboflavin	3.15	2.33	4.68	5.48	5.97
Niacin	2.46	1.72	3.68	5.06	4.66
Vitamin B_6_	5.74	4.40	9.78	7.93	8.84
Folate	4.87	3.53	9.12	6.48	8.91
Vitamin C	23.65	17.78	42.15	28.96	45.94
Vitamin A	2.44	1.47	3.70	6.76	4.03
Vitamin E	5.53	4.16	9.23	8.57	10.82

## Data Availability

Data are available at the Department of Food Market and Consumption research due to annual agreements signed between the Institute of Human Nutrition, Warsaw University of Life Sciences and Statistics Poland in Warsaw.
